# Parkinson’s disease-associated shifts between DNA methylation and DNA hydroxymethylation in human brain in PD-related genes, including PARK19 (DNAJC6) and PTPRN2 (IA-2β)

**DOI:** 10.21203/rs.3.rs-4572401/v1

**Published:** 2024-07-15

**Authors:** Juliana I. Choza, Mahek Virani, Nathan C. Kuhn, Marie Adams, Joseph Kochmanski, Alison I. Bernstein

**Affiliations:** Rutgers University; Rutgers University; Michigan State University; Van Andel Research Institute; Michigan State University; Rutgers University

**Keywords:** Epigenetics, Epigenomics, Parkinson disease, DNA methylation, PTPRN2, DNAJC6

## Abstract

**Background:**

The majority of Parkinson’s disease (PD) cases are due to a complex interaction between aging, genetics, and environmental factors; epigenetic mechanisms are thought to act as important mediators of these risk factors. While multiple studies to date have explored the role of DNA modifications in PD, few focus on 5-hydroxymethylcytosine (5hmC). Because 5hmC occurs at its highest levels in the brain and is thought to be particularly important in the central nervous system, particularly in the response to neurotoxicants, it is important to explore the potential role of 5hmC in PD. This study expands on our previously published epigenome-wide association study (EWAS) performed on DNA isolated from neuron-enriched nuclei from human postmortem parietal cortex from the Banner Sun Health Research Institute Brain Bank. The study aimed to identify paired changes in 5hmC and 5mC in PD in enriched neuronal nuclei isolated from PD post-mortem parietal cortex and age- and sex-matched controls. We performed oxidative bisulfite (oxBS) conversion and paired it with our previously published bisulfite (BS)-based EWAS on the same samples to identify cytosines with significant shifts between these two related epigenetic marks. Interaction differentially modified cytosines (iDMCs) were identified using our recently published mixed-effects model for co-analyzing β_mC_ and β_hmC_ data.

**Results:**

We identified 1,030 iDMCs with paired changes in 5mC and 5hmC (FDR < 0.05) that map to 695 genes, including *PARK19* (DNAJC6), a familial PD gene, and *PTPRN2* (IA-2), which has been previously implicated in PD in both epigenetic and mechanistic studies. The majority of iDMC-containing genes have not previously been implicated in PD and were not identified in our previous BS-based EWAS.

**Conclusions:**

These data potentially link epigenetic regulation of the *PARK19* and *PTPRN2* loci in the pathogenesis of idiopathic PD. In addition, iDMC-containing genes have known functions in synaptic formation and function, cell cycle and senescence, neuroinflammation, and epigenetic regulation. These data suggest that there are significant shifts between 5mC and 5hmC associated with PD in genes relevant to PD pathogenesis that are not captured by analyzing BS-based data alone or by analyzing each mark as a distinct dataset.

## Background

An estimated 5–10% of Parkinson’s disease (PD) cases are familial and caused by monogenically inherited mutations, while the remaining ~ 90% of sporadic cases (sPD) are likely due to a complex interaction between age, genes, and environmental factors (1–6). While the relative contribution of genetic and environmental risk factors in the etiology of sPD is debated, it is well documented that they play critical roles in the large majority of PD cases. Epigenetic mechanisms have emerged as critical mediators of the complex interactions between aging, genetics, and the environment because they are dynamic with age, sensitive to the environment, and regulate gene expression throughout the lifespan (7–12). Evidence for the role of epigenetic regulation in PD has been building, particularly for DNA modifications (12–20).

5-methylcytosine (5mC), the addition of a methyl group to the 5′-carbon of cytosine, is one of the most well-studied epigenetic marks (21). 5-hydroxymethylcytosine (5hmC) is formed via oxidation of 5mC by ten-eleven translocation (TET) enzymes and is a stable, independent epigenetic mark that has its highest levels in the brain, recruits a distinct set of DNA binding proteins from 5mC, differs in its genomic distribution in the brain compared 5mC, and is enriched in transcriptionally active gene bodies in the nervous system, suggesting a specific regulatory role for 5hmC in the brain (22–31). Thus, 5hmC is now thought to be particularly important in gene regulation in brain, particularly in the response to neurotoxicants (21, 24, 32, 33). However, most studies of DNA modifications in PD brain have relied on bisulfite (BS) conversion, which cannot distinguish between 5mC and 5hmC (12, 34–43).

Recently, studies have begun to explore links between 5hmC and PD (44–46). First, rare variants in *TET1* were associated with increased risk of PD in a Chinese PD cohort (44). Second, a targeted analysis of DNA modifications within known enhancers in human postmortem prefrontal cortex identified epigenetic disruption of an enhancer targeting the *TET2* gene in PD patients (46). This study also performed hydroxymethylated DNA immunoprecipitation-sequencing (hMeDIP-Seq) and found that PD-associated-hydroxymethylated peaks were enriched in gene bodies, promoters, and enhancers. Third, a small study in human postmortem substantia nigra (SN) used hMe-Seal, a selective chemical labeling method, and identified thousands of differentially hydroxymethylated regions in genes related to CNS and neuronal differentiation, neurogenesis, and development and maintenance of neurites and axons, although the widespread neurodegeneration in the SN by the time of PD diagnosis complicates interpretation of these results (45). Regardless, taken together, these initial studies support a role for 5hmC in regulation of expression of genes important for PD pathogenesis and indicate that additional research is warranted.

In our previous study, we performed a neuron-specific epigenome-wide association study (EWAS) with the Illumina EPIC BeadChip array paired with BS conversion using enriched neuronal nuclei from human postmortem parietal cortex obtained from the Banner Sun Health Research Institute Brain Bank (47). We identified largely sex-specific PD-associated changes in DNA modification in 434 unique genes, including genes previously implicated in PD, including *PARK7* (DJ-1), *SLC17A6* (VGLUT2), *PTPRN2* (IA-2β), and *NR4A2* (NURR-1), as well as genes involved in developmental pathways, neurotransmitter packaging and release, and axon/neuron projection guidance. However, we could not differentiate between 5mC and 5hmC because we used BS.

Here, we report the results of an epigenome-wide analysis of 5hmC and 5mC in enriched neurons from PD brain using our recently proposed method for reconciling base-pair resolution 5mC and 5hmC data (48–50). This method considers 5mC and 5hmC as paired data since these marks are biologically and statistically dependent on each other. We utilized additional DNA isolated from the same neuron-enriched samples and performed oxidative BS (oxBS) conversion paired with the Illumina EPIC BeadChip array to specifically measure 5mC (51, 52). oxBS adds an oxidation step with potassium perruthenate (KRuO_4_) that specifically oxidizes 5hmC, forming 5-formylcytosine, prior to BS conversion. BS then deaminates 5mC only, but not 5-formylcytosine, such that C and 5hmC are read as thymine, while only the 5mC is read as cytosine, providing a readout of “true” methylation. Comparison of BS and oxBS results allows estimation of 5hmC. To our knowledge, this is the largest epigenome-wide analysis of 5hmC to date in neurons enriched from PD post-mortem brain at single base pair resolution.

## Methods

### Human brain tissue

De-identified tissue samples from control (n = 50) and sPD (n = 50) human brain samples were obtained from archival human autopsy specimens provided by the Banner Sun Health Research Institute (BSHRI), using BSHRI’s approved institutional review board (IRB) protocols. Further details about the BSHRI’s brain samples and sample selection are available in a previous publication (53). We selected PD patients with mid-stage disease (Braak stage = II–III), as defined by Lewy pathology (54). Postmortem parietal cortex was selected because this region develops pathology in the late stages of PD and is expected to still have robust populations of neurons from which disease-associated, pre-degenerative gene regulatory marks can be measured.

### Magnetic-activated cell sorting

DNA samples previously isolated from an enriched neuronal population from de-identified parietal cortex samples from the BSHRI Brain Bank were used for this study. NeuN-positive (NeuN^+^) nuclei were enriched from 100 mg of flash-frozen parietal cortex tissue using a two-stage magnetic-assisted cell sorting (MACS) method as previously described (47).

### DNA extraction

DNA was isolated from enriched NeuN^+^ nuclei using the Qiagen QIAamp DNA Micro Kit (Cat. # 56304) as previously described (47).

### Oxidative bisulfite treatment and EPIC arrays

Intact genomic DNA yield was quantified by Qubit fluorometry (Life Technologies). oxBS conversion was performed on 1 μg genomic DNA using the TrueMethyl Array kit (Cambridge Epigenetix). While the recommended amount of 500 ng DNA was sufficient for BS conversions, this was insufficient for oxBS, likely due to the additional harsh oxidation step. Cleanup and preparation of DNA, and all steps for the EPIC bead chip protocol were performed as previously described per the manufacturer’s protocol (47).

### EPIC array data processing of oxBS data

IDAT files were imported into R and processed using an in-house bioinformatics pipeline that utilizes *minfi* (version 1.48.0), *ChAMP* (version 2.32.0), *posibatch* (version 1.0), and *ENmix* (version 1.38.01), and *CETS* (version 3.03) packages in R, as previously described (Supplementary File 1) (47, 48, 52, 55–62). After QC, eleven female samples and six male samples were removed due to a high level (> 10%) of failed probes, leaving 57 male (28 PD, 29 control) and 26 female (13 PD, 13 control) samples. Failed probes (53,305) were removed from remaining samples when detection p-value was > 0.01 in more than 5% of samples. Cross-reactive probes and probes containing SNPs (93,527) were masked based on previous identification (63).

We continued with male samples only due to small sample size of the remaining female samples. Samples with estimated glial cell proportion > 0.50 were also removed from analysis. This removed one sample from the male data set, leaving 56 males (27 PD, 29 control). Data for the included samples are summarized in Table 1 and full metadata is in Supplementary File 3.

**Figure 1:**
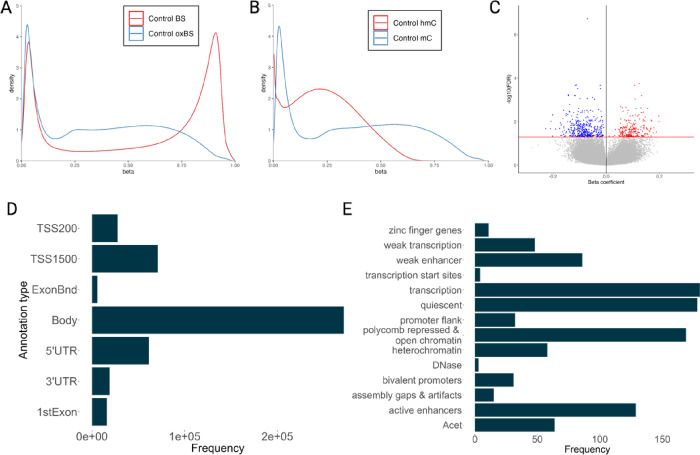
Visualization of β-values of selected PD-associated DMCs. (A-F) MLE-corrected β values graphed by modification (top) and raw BS β values with MLE-corrected β values graphed by disease status (bottom) for the most significant iDMC with a positive interaction term (A), the most significant iDMC with a negative interaction term (B), the iDMC with the most positive interaction term (C), the iDMC with the most negative interaction term (D) and the iDMC annotated to the PD gene *PARK19/DNAJC6* (E) and to *PTRPN2* (F). The corresponding beta coefficient/interaction term and FDR value are indicated for each iDMC.

5hmC β values (β_hmC_) were estimated by pairing oxBS β values with our previously published BS data using the maximum likelihood estimate function (oxBS.MLE) from the *ENmix* package, which returns true methylation β values (β_mC_) and estimates β_hmC_. Raw BS and oxBS values, as well as MLE-corrected β_mC_ and β_hmC_ values, are shown in Fig. 1A,B.

After MLE, probes with mean β_mC_ or β_hmC_ < 0.01 across all samples were removed due to increased variability and decreased interpretability of β values at such low levels, as well as to remove the issue of zero inflation for 5hmC β values. We then produced three data sets: one with all probes where β_mC_ > 0.01 (714,199 probes), one with all probes where β_hmC_ > 0.01 (588,147), and one with only probes common to the two sets, where β_mC_ > 0.01 and β_hmC_ > 0.01 (588,123). Of these, only 12 probes had β_hmC_ data only, and 126,076 had β_mC_ data only.

### Differential testing for individual CpG sites

The *gamlss* R package (version 5.4–22) was used to test for interaction differentially methylated cytosines (iDMCs) as previously described (Supplementary File 2) (47, 48, 64, 65). All models included glial cell proportion estimated from BS data and PMI as covariates (47). An FDR < 0.05 was used as the cutoff for significance and annotation of significant differential probes was performed using the Illumina EPIC array manifests. P-value histograms generated to confirm accuracy of modeling show that the interaction model provided more appropriate histograms (uniform with an overabundance of low p-values), whereas the separate models produced histograms with an overabundance of high p-values (Supplementary Fig. 1) (66). For separate analyses of β_mC_ and β_hmC_, neither normal nor beta regression modeling accurately modeled the data. All code for data visualizations is provided in Supplementary Files 6,7.

### Annotation of interaction DMCs

Gene IDs corresponding to each iDMC were extracted from the EPIC array manifest provided by Illumina (v1.0 B5). Annotation of universal chromatin states was also performed using the *annotatr* R package (version 1.28.0) and adding custom full stack ChromHMM chromatin states for *hg38* to the annotation cache (annotatr citation) (67, 68). Since this study utilized neuronally enriched nuclei, neuronal expression of iDMC-containing genes was verified using the Allen Brain Cell Atlas (69). The Harmonize and GeneCards databases were used to explore gene function (70, 71)

### Gene ontology pathway enrichment and protein-protein interaction networks

Gene ontology (GO) term enrichment testing and pathway analysis was performed on genes annotated to iDMCs identified in the interaction model using the ClueGO application in Cytoscape (version 3.10.1) (72, 73). “Groups” was selected as the visual style, and the GO biological process (GOBP) term was selected. Network specificity was set to “Medium”, with the GO Tree Interval minimum set at 3 and maximum at 8. Only terms with at least 3 genes and a Bonferroni-corrected p-value < 0.05 were included in pathway visualizations. The connectivity score (Kappa) was set at 0.4, and default GO Term Grouping settings were used in all analyses. The genes found in enriched GOBP terms by ClueGO were used for protein-protein interaction network analysis using STRING (version 12.0) (74). STRING network analysis was performed using default parameters, including a minimum required interaction score = 0.9 (Fig. 3) or 0.7 (Supplementary Fig. 2).

### Reanalysis of BS data

Data from our previous publication were reanalyzed to include intergenic loci to utilize updates to the EPIC manifest and include chromatin state annotation as performed here (47). This was carried out as previously described with the following modifications. The final filtering step to remove probes without genic annotations based on the Illumina EPIC array manifest was removed so that all probes that passed QC and filtering steps were included in the differential methylation analysis. Differentially methylated probes and differentially methylated regions were annotated to chromatin states by using the *annotatr* R package (version 1.28.0) and adding custom full stack ChromHMM chromatin states for *hg38* to the annotation cache (67, 68). Most of the DMCs, DMRs, and genes identified overlap with the results from our previous publication with some additions (Table 2). Annotated DMCs and DMRs identified here are included in Supplementary Files 9 and 10. Code for QC was run as previously written and modified code for the differential methylation analysis is provided in Supplementary File 8.

## Results

We identified 1,030 iDMCs with significant shifts in the proportions of 5mC and 5hmC associated with PD in DNA isolated from an enriched neuronal population derived from parietal cortex (FDR < 0.05) (Fig. 1C; Supplementary File 4). More iDMCs (652) have a negative than a positive beta coefficient (378). Consistent with known distribution of 5hmC, the majority of iDMCs were found in gene bodies (Fig. 1D). Chromatin state annotation shows that iDMCs are located mainly in transcriptionally active chromatin, consistent with their location within gene bodies, as well as quiescent chromatin, polycomb repressed and open chromatin, active enhances (Fig. 1E).

**Figure 2:**
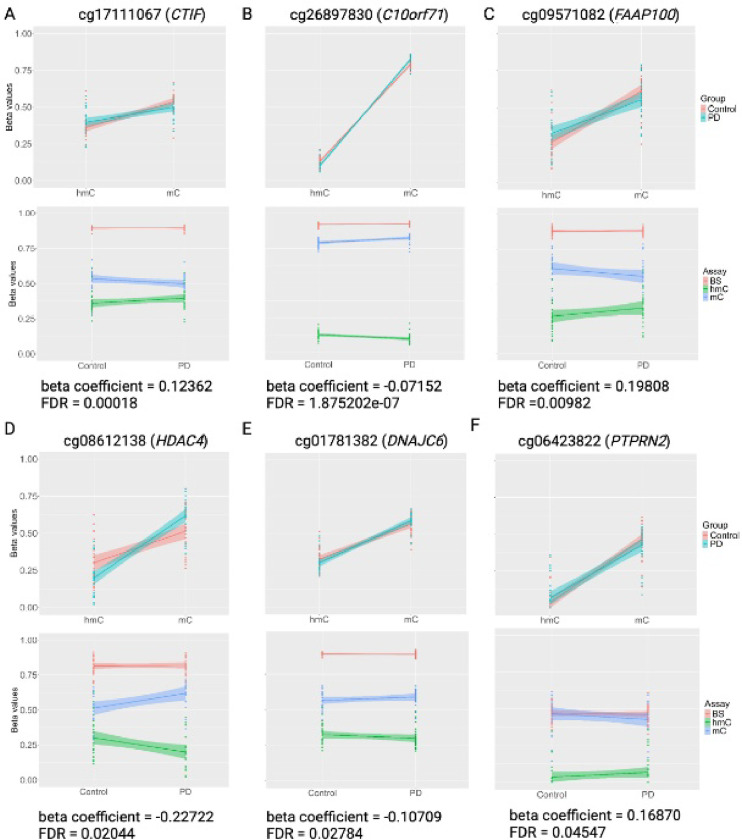
Visualization of β-values of selected PD-associated DMCs. (A-F) MLE-corrected β values graphed by modification (top) and raw BS β values with MLE-corrected β values graphed by disease status (bottom) for the most significant iDMC with a positive interaction term (A), the most significant iDMC with a negative interaction term (B), the iDMC with the most positive interaction term (C), the iDMC with the most negative interaction term (D) and the iDMC annotated to the PD gene *PARK19/DNAJC6* (E) and to *PTRPN2* (F). The corresponding beta coefficient/interaction term and FDR value are indicated for each iDMC.

In this output, a positive beta coefficient (interaction term) indicates a relative decrease in 5mC and an increase in 5hmC in PD brains, as illustrated by the most significant iDMC with a positive beta coefficient (Fig. 2A) and the DMC with the most positive beta coefficient (Fig. 2C). In contrast, a negative interaction term indicates a relative increase in 5mC and a decrease in 5hmC in PD brains, as illustrated by the most significant DMC with a negative beta (Fig. 2B) coefficient and the most negative beta coefficient (Fig. 2D). Corresponding raw BS β-values for each iDMCs demonstrate that these probes were not identified by BS data alone (Fig. 1F–J). Of these 1,030 iDMCs, 681 are found within genes and annotate to 695 genes, including the familial PD gene *PARK19* (DNAJC6) and *PTPRN2*, a gene previously implicated in PD and identified in multiple EWAS studies (Fig. 2E,F; Supplementary Files 5,12). Only 49 of these genes were also identified in our BS-only EWAS, but at different cytosines (Supplementary File 11) (47).

**Figure 3:**
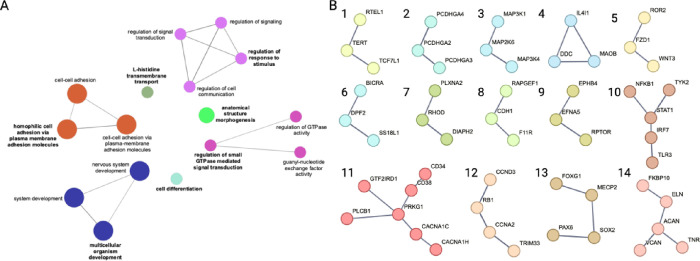
Gene ontology and protein interaction networks for DMC-containing genes. (A) Gene ontology network from ClueGO shows enriched groups of GO terms. Bolded text indicates the most significant GO term in each group. Grouped GO terms share at least 50% of their genes. (B) String protein interaction network for 374 unique genes (confidence < 0.9; >2 genes per network). All but 1 gene mapped to the STRING database (RTEL1-TNFRSF6B). PPI enrichment value = 5.91 × 10^−6^. Individual subnetworks are numbered for reference within the text.

Of the 695 genes, 375 annotated to 16 enriched GO terms in 7 GO term groups, which are enriched for genes involved in nervous system development, cell-cell adhesion, regulation of signal transduction and cell communication, morphogenesis, and cell differentiation (Fig. 3A; Table 3). Genes within the enriched GO terms were used for STRING network analysis to identify potential functional interactions between proteins encoded by differentially modified genes. All but 1 gene mapped to the STRING database (RTEL1-TNFRSF6B). For clarity, networks of proteins within enriched GO terms with the highest stringency settings are shown in Fig. 3B. Less stringent networks for each individual GO term group are shown in Supplementary Fig. 2 to highlight the high degree of potential connectivity within each group.

While there are major differences in existing PD EWAS related to sample size, sample selection, brain region assessed, methods used to measure DNA modifications, and statistical modeling, as well as inconsistent reporting between studies, we compared the list of genes in this study to recent brain-specific EWAS studies for PD, including ours, for which data was provided (36, 37, 40, 46). One study performed hMe-DIP in prefrontal cortex to assess genome-wide 5hmC (46). In addition, three BS-based studies used either the EPIC array or the previous 450K array in SN, frontal cortex, the dorsal motor nucleus of the vagus (DMV), and cingulate gyrus (CG) (36, 37, 40). Not surprisingly, given the differences between these studies, overlap between our data and these studies was minimal (Table 4, Supplementary File 12). The gene most consistently identified in these studies is *PTPRN2*, which we previously reported as showing sexually dimorphic alterations in DNA modifications and has previously been implicated in PD (47, 75–77). The methodological differences between these studies complicate the comparison of these studies and underscore the need for rigorous and reproducible methodology and analysis, as discussed in our recent chapter on rigor and reproducibility in EWAS (78).

## Discussion

### Significant shifts between 5mC and 5hmC associated with PD

Here, we performed an integrated genome-wide analysis of 5mC and 5hmC using our novel application of mixed effects modeling in enriched neuronal nuclei from PD post-mortem parietal cortex samples (48–50) These PD-associated iDMCs were largely unique from DMCs identified in our previous BS-based EWAS: 49 genes were identified in both studies, 646 only in the paired analysis, and 498 genes identified only in the BS-only analysis (Supplementary Files 5,9–11) (47). Collectively, these data suggest that there are significant PD-associated shifts between 5mC and 5hmC at iDMCs that are not captured by analyzing BS-based data alone or by analyzing each mark as distinct datasets (Fig. 1C) (47, 48). Because many of these iDMCs are located within genes previously implicated in PD and in genes within pathways thought to play a role in PD, these data suggest that shifts in the balance between DNA modifications may play an important but unrecognized role in PD etiology in both known and novel PD-related genes.

An important caveat of these findings is that this study does not address the biological significance of these epigenetic shifts. While shifts between 5mC and 5hmC may potentially impact the binding of proteins that regulate gene expression and/or other epigenetic marks, this study does not examine these functional impacts; however, it does provide multiple avenues for further study of the impact of these changes on gene expression, alternate promoter usage, differential isoform expression, and neuronal function and susceptibility.

### Epigenetic regulation of the familial PARK19 (DNAJC6) PD gene

As with our previous study, we identified epigenetic changes in known PD genes. Here, we identified an iDMC within the 3′ UTR of *PARK19*, which encodes DNAJC6. This iDMC shows increased 5mC and decreased 5hmC in PD and is located in a region annotated by universal chromatin state annotation as a transcribed enhancer (Fig. 2E; Supplementary File 5) (67). Autosomal recessive mutations in *PARK19* predominantly cause juvenile- or early-onset PD, with atypical signs and symptoms characterized by poor L-DOPA responsiveness, pyramidal signs, dystonia, rapid disease course, mental retardation, and seizures and is one of multiple DNAJC genes associated with familial forms of PD (2, 5, 79–83). DNAJC6 works with multiple other genes linked to familial and sporadic PD, including *PARK20* (SYNJ1), *PARK8* (LRRK2), *PARK2* (PRKN), *SNCA* (α-synuclein), and other DNAJC proteins (*DNAJC26*, RAK; *DNAJC13*, REM8), to regulate key steps in synaptic vesicle trafficking, synaptic vesicle endocytosis, and clathrin-dynamics in endocytosis (83–87). While mutations in this gene have only been associated with rare familial forms of PD, these data potentially link epigenetic regulation of this locus with the pathogenesis of sPD and are consistent with previous findings from our lab and others demonstrating epigenetic changes in familial and GWAS PD genes in brain tissue from sPD cases (12, 38–41, 47).

### Epigenetic regulation of PTPRN2

The gene most consistently identified in this and other EWAS studies is *PTPRN2* (Fig. 2F; Supplementary File 12). We previously reported sexually dimorphic alterations in DNA modifications within this gene, and all but one of the previous EWAS in our comparison also identified altered DNA modifications within this gene (36, 37, 40, 47). In our previous study, we found multiple DMCs and one DMR annotated to the *PTPRN2* gene that showed exact, complete overlap in both male and female samples and was hypermethylated in male samples but hypomethylated in female samples (47). *PTPRN2* encodes the protein tyrosine phosphatase receptor type N2 (IA-2β), which is a major autoantigen in Type 1 diabetes and is expressed on dense core and synaptic vesicles. DNA modifications within this locus have also been associated with Type 2 diabetes and pesticide exposures (88, 89). In addition to its well-established role in insulin release and diabetes, multiple studies demonstrate a role for this gene in the release of monoamines (dopamine, norepinephrine, and serotonin) in the brain that lead to deficits in learning and memory, and motor behavior (75, 90–92). Specifically relevant to PD, a hypomethylated DMC within *PTPRN2* in whole blood was associated with faster motor progression (77). In addition, decreased expression of *PTPRN2* has also been observed in the SN of PD patients, and increased expression has been seen in DA neurons derived from PD patients with *LRRK2* G2019S mutations (75, 76). Mechanistically, there are multiple non-coding transcripts, alternate protein-coding transcriptions, and splice variants encoded by the *PTPRN2* locus, and expression of *PTRPRN2* is regulated by multiple miRNAs (93, 94). Thus, epigenetic regulation of this locus could potentially lead to altered transcript usage or differential regulation by miRNAs that affect monoaminergic signaling.

#### PD-associated interaction DMCs are enriched in pathways related to synaptic formation and function, senescence, neuroinflammation, and epigenetic regulation

Within the 695 iDMC-containing genes, our functional annotation found that these gene products function within specific pathways related to neuronal dysfunction in PD (Fig. 2, Table 2). These pathways are described here with STRING subnetworks in Fig. 2B indicated by their numbers; expanded connections with additional iDMC-containing genes are shown in Supplementary Fig. 2.

Multiple iDMC-containing genes encode proteins that converge on functions related to proper development and function of synapses, including synaptogenesis, synaptic plasticity, neurotransmitter release, and clathrin-mediated endocytosis (networks 2,4,7,8,9,10,13,14) (Fig. 2B). In addition, a set of genes play roles in the myelination and maintenance of myelin (networks 3,5), as well as in the synthesis of dopamine, serotonin, and norepinephrine (network 4) (Fig. 2B). Together, this suggests the regulation of genes related to the formation of synapses and the maintenance of synaptic and vesicular integrity are disrupted prior to the onset of pathology within the parietal cortex. This is also consistent with findings in our mouse model of increased PD susceptibility, showing that exposure to the organochlorine pesticide dieldrin is associated with epigenetic changes in similar key neurodevelopmental pathways across the lifespan (95). Together, these pathways coordinate the formation, structure, function, and plasticity of synapses, consistent with the idea that the integrity of synapses is critical for proper neurotransmission and that multiple PD-related mechanisms converge on the disruption of synaptic function (96–99).

A large body of evidence now supports a role of neuroinflammatory pathways in PD (100–102). Here, we identified multiple iDMC-containing genes important in the neuronal response to environmental stressors and neuroinflammatory signals, including multiple genes within the NFκB signaling pathway (networks 3,11) (Fig. 2B). Altered epigenetic regulation of these genes could lead to impropriate activation of these pathways in response to cellular and environmental stressors. We also identified genes involved in cell cycle and senescence pathways (1, 12), consistent with evidence that neuronal cell cycle reentry events and cellular senescence in the aging brain can lead to degeneration (103–105). Finally, we observed epigenetic regulation of multiple epigenetic regulators themselves (network 6) (Fig. 2B).

While these changes were not assessed in the SN, the region most commonly studied in the context of PD, by using the parietal cortex, which does not have widespread degeneration, we were able to assess neuron-specific DNA modifications associated with PD in a region without widespread neuron loss prior to the onset of pathology within the parietal cortex. Despite this, overall, these data suggest that PD-associated alterations in the epigenetic regulation of these genes may alter gene expression, promoter usage, or isoform expression of these genes and may represent early events that precede the onset of degeneration.

### Technical limitations of the oxBS method

In addition, it is also important to note that this study included a high level of failed probes and samples that was unique to the oxBS reactions. In contrast, no samples were excluded due to high levels of failed probes, and far fewer probes failed from the BS data. As noted in the methods, we started with 1 μg of input DNA for oxBS because when we used the recommended starting amount of 500 ng, all oxBS probes failed. Together, this suggests that oxBS is much harsher on the DNA, requiring high input amounts that may limit the utility of this method as the field moves towards cell type-specific methods and lower input amounts.

## Conclusions

These results draw a possible link between epigenetic regulation of the *PARK19* and *PTPRN2* loci and the pathogenesis of sPD. Additional iDMC-containing genes play critical roles in synaptic formation and function, cell cycle and senescence, neuroinflammation, and epigenetic regulation. This is also consistent with findings in our mouse model of increased PD susceptibility, showing that exposure to the organochlorine pesticide dieldrin is associated with epigenetic changes in similar pathways across the lifespan (95). Together, these pathways coordinate the formation, structure, function, and plasticity of synapses, consistent with the idea that the integrity of synapses is critical for proper neurotransmission and that multiple PD-related mechanisms converge on the disruption of synaptic function (96–99). In addition, most of these genes were not identified by a BS-only analysis on the same samples, suggesting that there are significant shifts between 5mC and 5hmC associated with PD in genes relevant to PD pathogenesis that are not captured by analyzing BS-based data alone or by analyzing each mark as a distinct dataset.

## Figures and Tables

**Table 1:** Cohort characteristics of samples included in the analysis. Data includes disease status, age at death in years, postmortem interval (PMI) in hours, and race of samples remaining after QC. Seven male samples and eleven female samples were removed during quality control and pre-processing, leaving 56 male and 26 female samples.

	Male (n = 56)	
Variables	Mean ± SD or N (%)	Range
*Disease Status*		
Control	29 (51.8%)	
Parkinson’s disease	27 (48.2%)	
*Age at Death*		
Control	79.3 ± 9.1	53–93
Parkinson’s disease	79.4 ± 7.1	64–91
*PMI*		
Control	3.25 ± 0.81	2.16–5.5
Parkinson’s disease	3.27 ± 0.83	1.83–4.92
*Race*		
White	55 (98.2%)	
Unidentified	1 (1.8%)	

**Table 2:** Comparison of published and reanalyzed results of BS-only data.

	DMCs		DMRs		Genes		
	Male	Female	Male	Female	Male	Female	Total Unique
**Kochmanski 2022**	3	87	258	214	258	282	523
**BS Reanalysis**	4	117	267	223	261	303	547
**Overlap**	2	77	208	195	242	268	494

**Table 3:** Enriched GO Terms and GO Term Groups of interaction DMC-containing genes. Results of ClueGO geneontology enrichment analysis and significant GO terms are shown (p < 0.05). GO terms are grouped when they share > 50% of their genes. “Associated Genes” shows all interaction DMC containing genes that map to each GO Term Group.

GO Groups	GO Terms	Associated Genes Found
Group0	L-histidine transmembrane transport	SLC15A4, SLC25A29, SLC38A3, SLC7A1
Group1	anatomical structure morphogenesis	ABCA2, ACAN, ACTN2, AGRN, ALPL, ALX4, ANO1, APOA1, ARHGEF15, ARX, ATP10A, BARX2, BCR, BMP1, CACNA1C, CACNA1H, CASQ1, CCBE1, CCDC141, CCNA2, CD34, CDH11, CDH13, CDH23, CDH4, CLRN2, CNMD, COL22A1, COL27A1, COL4A3, CSF1R, CSNK2B, CTSZ, DCHS1, DDR1, DIO3, DISC1, DLL1, DNM3, DOCK2, DSCAML1, EDAR, EFNA5, EGFLAM, EIF2AK4, ELN, EMX1, EPHB4, ETS1, F11R, FAM20C, FAT3, FBXW8, FGFR2, FKBP10, FOXG1, FRMD6, FZD1, GAA, GAS7, GLI2, GNAS, HAND1, HGS, HOXA3, HS3ST3B1, HSBP1, IGF2BP2, INF2, IRX2, ITGB5, KCNQ1, KDM5B, LAMA2, LIMD1, LRIG3, LTBP3, LYVE1, MAP2K5, MAP3K13, MBP, MECP2, MRTFA, MYH3, MYMK, MYOD1, NCAM1, NFKB1, NYAP1, OBSCN, OTX1, PAX6, PCDHGB4, PLEKHA1, PLXNA2, PLXND1, POFUT1, PPFIA2, PRAG1, RAB23, RADIL, RB1, RERE, RFX2, RHOD, RIPOR2, ROR2, SASH1, SDK1, SKI, SMOC2, SOX15, SOX2, SP100, SPARC, SS18L1, ST6GAL1, STAB1, STAT1, STK24, SYT2, TAFAZZIN, TERT, TFAP2A, TGFB1, TIAM2, TLR3, TMEM100, TNFSF11, TNR, TOPORS, TSPEAR, VAV2, VSX1, WNT3, WNT9A, WWOX, ZFP36L1
Group2	cell differentiation	ABCA2, ACAN, ACTN2, AGRN, AKNA, ALPL, ANO1, APC2, APOA1, ARX, ASAP1, ATP11A, BARX2, BCR, BFSP1, BICRA, BMP1, BPGM, BRINP2, BTBD2, C9orf24, CACNA1H, CASQ1, CCDC141, CCNA2, CD34, CD38, CDH1, CDH11, CDH23, CDH4, CDYL, CEBPE, CEP85L, CFAP157, CLRN2, CNMD, COL22A1, COL27A1, CSF1R, CSF3R, CSK, DCHS1, DDR1, DIAPH2, DIO3, DISC1, DLK1, DLL1, DNASE1L3, DNM3, DOCK2, DPF2, DSCAML1, DYSF, EDAR, EFNA5, EID2B, EIF2AK4, EMX1, EPHB4, ERG, ETS1, F11R, F2RL1, FAM20C, FAT3, FBXW8, FCER1G, FGFR2, FOXG1, FOXO6, FRMD6, FZD1, GAK, GAS7, GCNT2, GLI2, GPAT4, GRM5, HAND1, HDAC4, HSBP1, IL18R1, IL4I1, IRF4, IRF7, IRX2, ITGA11, ITGB5, KCNQ1, KDM5B, KRT8, LAMA2, LDLRAD4, LIMD1, LTBP3, MAP2K6, MAP3K13, MAP3K4, MBP, MECP2, MLH1, MMD2, MRTFA, MTURN, MYH3, MYMK, MYOD1, NAV2, NCAM1, NEU4, NFKB1, NFKBID, NHSL2, NKX6–3, NPHP4, NTM, NTRK3, NUMA1, NXN, NYAP1, OBSCN, OSBP2, PAX6, PIEZO1, PIWIL3, PLCB1, PLEKHA1, PLXNA2, PLXND1, POU6F2, PPFIA2, PRAG1, PRDM16, PRKG1, PRLR, PRM2, PRM3, PRRC2A, PRRC2C, RAB32, RADIL, RAPGEF1, RB1, RERE, RFX2, RIPOR2, ROR2, RTN4RL1, SCUBE1, SDK1, SKI, SLC12A5, SLC4A5, SLCO4C1, SMOC1, SOCS2, SOX15, SOX2, SPAG16, SS18L1, ST6GAL1, STAT1, STK24, SYNE2, SYT2, TBX21, TBX22, TCF7L1, TERT, TESMIN, TFAP2A, TGFB1, TIAM2, TLR3, TMEM100, TMEM91, TNFSF11, TNR, TOPORS, TP73, TRAPPC9, TST, TYK2, VCAN, VSX1, WDR38, WNT3, WNT9A, WWOX, ZBTB7B, ZFP36L1
Group3	multicellular organism development, system development, nervous system development	AATK, ABCA2, ACAN, ACTN2, AGRN, AK8, AKNA, ALPL, ALX4, ANKLE2, ANO1, APBA3, APC2, APOA1, ARHGEF15, ARNT2, ARX, ASAP1, ATN1, ATP11A, BARX2, BCR, BFSP1, BMP1, BPGM, BRINP2, BTBD2, CACNA1C, CCBE1, CCDC141, CCNA2, CD34, CD38, CDH1, CDH11, CDH13, CDH23, CDH4, CEBPE, CEP85L, CLRN2, CNMD, COL22A1, COL27A1, COL4A3, CRYGN, CSF1R, CSF3R, CSK, CSNK2B, CTNS, CTSZ, DCHS1, DDC, DDR1, DHX35, DIO3, DISC1, DLL1, DNAJB1, DNM3, DOCK2, DPF2, DSCAML1, EDAR, EFNA5, EIF2AK4, ELN, EMX1, EPHB4, ETS1, F11R, F2RL1, FAM20C, FAT3, FBXW8, FCER1G, FGFR2, FKBP10, FOXG1, FOXO6, FZD1, GAA, GAK, GAS7, GCNT2, GLI2, GNAS, GRM5, HAND1, HDAC4, HGS, HOXA3, HS3ST3B1, HSBP1, HSD17B3, IGF2BP2, IL18R1, IL4I1, INPP5K, IRF4, IRF7, IRX2, ITGB5, KCNQ1, KDM5B, KIF26A, LAMA2, LRIG3, LTBP3, MAD1L1, MALL, MAOB, MAP2K5, MAP2K6, MAP3K13, MAP3K4, MBP, MECP2, MLH1, MMD2, MPST, MRTFA, MTURN, MYH3, MYOD1, NAV2, NCAM1, NEU4, NFKBID, NKX6–3, NPHP4, NRXN2, NTM, NTRK3, NUMA1, NXN, NYAP1, OTX1, PAX6, PCDHGA1, PCDHGA10, PCDHGA2, PCDHGA3, PCDHGA4, PCDHGA5, PCDHGA6, PCDHGA7, PCDHGA8, PCDHGA9, PCDHGB1, PCDHGB2, PCDHGB3, PCDHGB4, PCDHGB5, PCDHGB6, PDCD1, PDE6B, PDGFD, PKD1L1, PLCB1, PLCE1, PLEKHA1, PLXNA2, PLXND1, POFUT1, POU6F2, PPFIA2, PRAG1, PRDX1, PRKG1, PRLR, PRRC2C, RAB23, RAPGEF1, RB1, RBM19, RD3, RERE, RGS7, RIPOR2, ROR2, RTN4RL1, SASH1, SBF2, SCUBE1, SDK1, SKI, SLC12A5, SLC15A4, SLC34A2, SLC38A3, SLC4A5, SMOC1, SMOC2, SOCS2, SOX15, SOX2, SP100, SPARC, SS18L1, STAB1, STAT1, STK24, SYNE2, SYNJ2, SYT2, TAFAZZIN, TBX21, TCF7L1, TEAD1, TERT, TFAP2A, TGFB1, TIAM2, TLR3, TMEM100, TMEM91, TNFSF11, TNR, TOPORS, TP73, TRAPPC9, TRIM26, UMODL1, VAV2, VCAN, VSX1, WDR38, WNT3, WNT9A, WWOX, XYLT1, ZBTB7B, ZFP36L1
Group4	regulation of GTPase activity, regulation of small GTPase mediated signal transduction, guanyl-nucleotide exchange factor activity	ABR, AGAP1, AGRN, APC2, APOA1, ARHGAP42, ARHGAP45, ARHGAP6, ARHGEF15, ARHGEF4, ASAP1, BCR, BMP1, CGNL1, CHN2, DENND1A, DENND1C, DOCK2, DOCK6, EFNA5, F11R, F2RL1, GNAS, HERC2, INF2, MCF2L, MCF2L2, MMD2, NTRK3, NUP62, OBSCN, PLCE1, PLEKHG4B, PLEKHG7 PLXNA2, PLXND1, PRAG1, PRKG1, RAPGEF1, RGS7, RHOD, RIPOR2, SBF2, SGSM2, SLC6A5, TBC1D10B, TIAM2, VAV2
Group5	cell-cell adhesion, cell-cell adhesion via plasma-membrane adhesion molecules, homophilic cell adhesion via plasma membrane adhesion molecules	AKNA, APOA1, ARVCF, CCNA2, CD34, CD93, CDH1, CDH11, CDH13, CDH23, CDH4, CLDN10, CLDN2, CSK, DCHS1, DSCAML1, EFNA5, ELANE, ETS1, F11R, FAT3, GCNT2, GLI2, GNAS, IGDCC4, IL4I1, ITGA11, ITGB5, MAD1L1, MAP2K5, MBP, NFKBID, NPHP4, NRXN2, OBSCN, PCDHGA1, PCDHGA10, PCDHGA2, PCDHGA3, PCDHGA4, PCDHGA5, PCDHGA6, PCDHGA7, PCDHGA8, PCDHGA9, PCDHGB1, PCDHGB2, PCDHGB3, PCDHGB4, PCDHGB5, PCDHGB6, PIEZO1, PKD1L1, PKP3, PPM1F, PRKG1, RAPGEF1, RIPOR2, SDK1, SLC7A1, SOX2, TBX21, TGFB1, TNFSF11, TNR, TYK2, ZBTB7B, ZFP36L1
Group6	regulation of signaling, regulation of response to stimulus, regulation of cell communication, regulation of signal transduction	ABCB1, ABR, ADGRE2, AGPAT2, AKNA, ANO1, APC2, APOA1, ARHGAP42, ARHGAP45, ARHGAP6, ARHGEF15, ARHGEF4, BCR, C10orf71, CAMTA1, CASQ1, CCBE1, CCNA2, CCND3, CD34, CD38, CD59, CDH1, CDH11, CDH13, CDK14, CFB, CFD, CFHR5, CGNL1, CHN2, CIDEA, CLEC16A, CLPB, CRTC3, CSF1R, CSK, CSNK2B, CTNS, DAPK2, DENND1A, DISC1, DLGAP4, DLK1, DLL1, DNAJB1, DNASE1L3, DOCK2, DPF2, EDAR, EFNA5, EIF2AK4, ELANE, ETS1, F11R, F2RL1, FAM107A, FAM20C, FCER1G, FGFR2, FGFR4, FRMD8, FZD1, GCNT2, GCSAML, GLI2, GNAS, GRAMD4, GRID1, GRM4, GRM5, GTF2IRD1, HDAC4, HEBP2, HGS, IL18R1, IL4I1, INF2, INPP5K, IRAK2, IRF4, IRF7, JPH3, KCTD6, KDM5B, KIF26A, KLHL15, LAMA2, LDLRAD4, LIMD1, LRPAP1, MAD1L1, MAGI3, MAP2K5, MAP2K6, MAP3K1, MAP3K13, MAP3K4, MAP4K2, MCF2L, MECP2, MLH1, MMD2, MRTFA, MTURN, MYMK, MYOD1, NCAM1, NCOA7, NFKB1, NFKBID, NFKBIL1, NLRP7, NPHP4, NTRK3, NTSR1, NUMA1, NUP62, NXN, OBSCN, P2RY6, PDCD1, PDGFD, PFKL, PLCB1, PLCE1, PLEKHA1, PLEKHF1, PLEKHG4B, PMEPA1, POFUT1, PPM1F, PRAG1, PRDM16, PRDX1, PRKG1, PRLR, RAPGEF1, RB1, RGS7, RHOD, RIPOR2, RNF126, RNF185, ROR2, RPTOR, RTEL1-TNFRSF6B, RTEL1, RTN4RL1, SASH1, SCUBE1, SECTM1, SETD4, SHISA7, SIGIRR, SKI, SLC15A4, SLC6A5, SMOC2, SOCS2, SOX15, SOX2, SP100, SREBF2, SSTR5, ST6GAL1, STAT1, STK24, STK39, STX4, STXBP2, TBX21 TCF7L1, TEDC2, TERT, TGFB1, TIAM2, TLR3, TMEM100, TNFSF11, TNR, TP73, TRABD2B, TRAP1, TRIM33, TSPEAR, TYK2, UBR5, USP44, VAV2, VWF, WNT3, WWOX, ZBTB7B, ZFYVE28, ZNF366

**Table 4:** Summary of comparisons between the current study and previous PD EWAS in brain tissue.

	5hmC	5mC and 5hmC (BS)							Paired BS/oxBS
	Marshall 2020	Kia 2021		Masliah 2013	Young 2019			Kochmanski 2022	Current study
**Control (M/F)**	23*	not specified		6 (2/4)	41 (all male)			49 (29/20)	29 (all male)
**PD (M/F)**	20*	134*		5(5/0)	38 (all male)			50 (33/17)	27 (all male)
**Region**	PFC	SN	CTX	FC	DMV	CG	SN	PC	PC
**Method**	hMe-DIP	BS-450K		BS-450K	BS-450K/EPIC			BS-EPIC	BS/oxBS-EPIC
**# genes**	5157	154	125	155	203	119	1466	547	695
**Overlap with BS**	126	5	9	12	20	14	68	-	49
**Overlap with interaction**	173	6	11	14	20	8	105	49	-

* Sex not specified, Abbreviations: SN, substantia nigra; PFC, prefrontal cortex; CTX, cortex; FC, frontal cortex; DMV, dorsal motor nucleus of the vagus; CG, cingulate gyrus; PC, parietal cortex

## Data Availability

This study was preregistered with Open Science Framework: https://osf.io/z4vbw. Raw and processed data are available in GEO (GSE267937): https://www.ncbi.nlm.nih.gov/geo/query/acc.cgi?acc=GSE267937 All supplementary material, including additional figures, tables of results, and code used for analyses, are available as supplementary files.

## Abbreviations

5hmC: 5-hydroxymethylcytosine
5mC: 5-methylcytosine
BS: bisulfite treatment
BSHRI: Banner Sun Health Research Institute
CG: cingulate gyrus
DMC: differentially modified cytosine
DMV: dorsal motor nucleus of the vagus
EWAS: epigenome-wide association study
GO: gene ontology
GOBP: GO biological process
GWAS: genome-wide association study
hMeDIP-Seq: hydroxymethylated DNA immunoprecipitation-sequencing
iDMC: interaction differentially modified cytosine
MLE: maximum likelihood estimate
oxBS: oxidative bisulfite treatment
PD: Parkinson’s disease
PMI: postmortem interval
SN: substantia nigra
SNP: single nucleotide polymorphism
sPD: sporadic Parkinson’s disease
TET: ten-eleven translocase
UTR: untranslated region
